# The Liver as the Central Regulator of Cholesterol Homeostasis: Statins, Gut Microbiota, Hepatic Inflammation, and the Proposed Oral–Gut–Liver–Artery Axis in Atherogenesis

**DOI:** 10.3390/metabo16070495

**Published:** 2026-07-13

**Authors:** Mark Cannon, John Peldyak, Eleanor Campbell

**Affiliations:** 1 Feinberg School of Medicine, Northwestern University, Chicago, IL 60611, USA; 2 Private Practice, Mount Pleasant, MI 48858, USA; njjn@ispmgt.com; 3 Department of Family Medicine, Philadelphia College of Osteopathic Medicine (PCOM), Philadelphia, PA 19131, USA; dr@campbellfamilymedicine.com

**Keywords:** cholesterol homeostasis, liver, statins, statin-associated muscle symptoms, gut microbiota, bile acids, *Porphyromonas gingivalis*, phosphorylated dihydroceramides, sphingolipids, bacterial ceramides, Bacteroidetes-derived lipids, serine dipeptide lipids, ceramide, spatial lipidomics, metabolic dysfunction-associated steatotic liver disease, atherosclerosis, viridans streptococci

## Abstract

**Background/Objectives:** Cholesterol homeostasis is often framed as a dietary problem, but circulating low-density lipoprotein (LDL) biology is governed largely by endogenous sterol handling, with the liver acting as the principal integrative organ for cholesterol synthesis, LDL receptor-mediated clearance, very-low-density lipoprotein (VLDL) export, bile acid production, and biliary sterol disposal. This narrative review evaluates the hepatic basis of cholesterol regulation, statin pharmacology, gut microbial sterol metabolism, chronic hepatic inflammation, and a proposed oral–gut–liver–artery axis in atherogenesis. The aim of this narrative review is to clarify which elements of the proposed axis are established, which are supported but incomplete, and which remain hypothesis-generating. **Methods:** Mechanistic, translational, clinical, and review literature were synthesized to separate established mechanisms from emerging and speculative links. PubMed/MEDLINE, Scopus, and Google Scholar were searched from January 2000 through May 2026. Primary search terms included: cholesterol homeostasis, LDL receptor, SREBP2, statin pleiotropic effects, statin-associated muscle symptoms, gut microbiota cholesterol, bile salt hydrolase, MASLD, *Porphyromonas gingivalis* liver, phosphorylated dihydroceramides, serine dipeptide lipids Bacteroidetes, ceramide atherosclerosis, and oral–gut–liver–artery axis. **Results:** LDL/apoB causality and hepatic statin mechanism are well-established. Gut microbiota can alter cholesterol absorption, coprostanol formation, bile acid pools, and portal signaling, but these effects are context-dependent. Hepatic free cholesterol loading and lysosomal sterol stress are strongly implicated in the biology of metabolic dysfunction-associated steatotic liver disease (MASLD). Periodontal pathogens, especially *Porphyromonas gingivalis*, may contribute to liver and vascular inflammation through bacteremia, oral–gut translocation, innate immune activation, and bioactive bacterial sphingolipids. Phosphorylated dihydroceramides (PDHCs) and Bacteroidetes-derived serine dipeptide lipids have been detected in human arterial specimens and shown to enter host ceramide pools, providing a direct lipid metabolic pathway linking microbial community composition to vascular disease. Viridans streptococci and the *Streptococcus anginosus* group are inflammatory cofactors rather than proven causes of hepatic cholesterol overproduction. **Conclusions:** The strongest model involves microbial amplification of hepatic cholesterol dysmetabolism, endothelial activation, foam-cell formation, and plaque vulnerability acting on a host-derived LDL/apoB scaffold. This model is testable and should complement guideline-based LDL-lowering therapy.

## 1. Introduction

Atherosclerotic cardiovascular disease is driven by the retention of apoB-containing lipoproteins within the arterial wall, and LDL is now supported as a causal risk factor by genetic, epidemiologic, and interventional evidence [1,2]. However, clinical and public discussions often overemphasize dietary cholesterol while underemphasizing endogenous sterol synthesis and hepatic regulation. This distinction is clinically relevant because the liver integrates the dominant control points for plasma LDL: sterol sensing, LDL receptor expression, cholesterol synthesis, VLDL secretion, bile acid synthesis, and biliary sterol excretion [3,4].

This does not mean that the liver is the only organ that synthesizes cholesterol. Cholesterol is produced by many nucleated cells, including intestinal, adrenal, immune, and neural cells. The liver is nevertheless positioned uniquely at the interface of portal nutrient flow, lipoprotein clearance, lipoprotein export, bile acid metabolism, and innate immune surveillance. It therefore exerts disproportionate influence over circulating LDL concentrations and atherogenic flux [5,6].

A second theme is immunometabolism. Cholesterol flux shapes lipid-raft signaling, inflammasome activation, macrophage phenotype, antigen presentation, and oxysterol signaling; inflammatory cytokines, in turn, alter sterol synthesis, trafficking, efflux, and lysosomal handling [7]. The liver is therefore not merely a biochemical factory but an immunometabolic hub in which lipid regulation and inflammation can reinforce one another.

A biologically plausible, though not yet proven, possibility is that chronic oral and enteric microbial exposures increase hepatic cholesterol retention, alter the handling of atherogenic lipoproteins (ApoB-containing), and contribute to arterial inflammation. Periodontal pathogens gain systemic access via recurrent bacteremia and endotoxin exposure, triggering inflammatory signaling that alters hepatic lipid metabolism and promotes the overproduction of VLDL and its downstream derivative, LDL. Oral pathogens appear to act through both direct atherogenic mechanisms and indirect inflammatory amplification, linking hepatic lipid handling and arterial disease within a unified host–microbe framework [8].

The present review advances a deliberately conservative hypothesis. The aim of this review is to clarify which parts of this model are proven, which are supported but incomplete, and which remain hypothesis-generating.

## 2. Scope and Narrative Synthesis Approach

This manuscript is a narrative review and hypothesis-generating synthesis rather than a systematic review or meta-analysis. Literature was identified by searching PubMed/MEDLINE, Scopus, and Google Scholar. The primary search covered January 2000 through May 2026; selected foundational references from earlier periods were retained where no more recent primary study superseded them. Primary search terms included: cholesterol homeostasis, LDL receptor, SCAP, SREBP2, HMG-CoA reductase, statin mechanism, statin pleiotropic effects, statin-associated muscle symptoms, gut microbiota cholesterol, bile salt hydrolase, coprostanol, bile acids atherosclerosis, MASLD NAFLD free cholesterol, *Porphyromonas gingivalis* liver NAFLD, phosphorylated dihydroceramides, serine dipeptide lipids Bacteroidetes, ceramide cardiovascular, spatial lipidomics plaque, viridans streptococci bacteremia, *Streptococcus anginosus*, and oral–gut–liver–artery axis. Reference lists of the included reviews were also hand-searched for additional primary studies.

The synthesis prioritizes primary mechanistic studies, human cohort data, consensus statements, randomized-trial meta-analyses, and recent review-level evidence. Causal language is deliberately qualified when the evidence is associative, animal-based, or based on microbial detection rather than proven mechanism. Confidence categories in Table 1 (Established, Moderate, Emerging, Weak) represent the authors’ qualitative assessment of the cumulative weight of evidence based on consistency across study types, replication across independent groups, availability of mechanistic data, and evidence from human versus animal systems. These are narrative judgments, not formal GRADE ratings.

## 3. Liver-Centered Cholesterol Homeostasis and Statin Pharmacology

### 3.1. The Liver as the Principal Regulator of Circulating Cholesterol

Hepatocyte sterol sensing is organized around the SCAP–SREBP2 system. When intracellular cholesterol is depleted, SREBP2 activation increases the transcription of genes involved in cholesterol synthesis and uptake, including HMGCR and LDLR. When sterol abundance rises, SCAP retention suppresses this transcriptional program [3,4]. This feedback architecture explains why plasma LDL often reflects hepatic sterol balance more than dietary cholesterol intake alone.

Hepatic cholesterol can be retained in membranes, esterified for storage or VLDL assembly, secreted into plasma lipoproteins, converted to bile acids, or excreted through biliary transport. SREBP2, liver X receptors, farnesoid X receptor signaling, ATP-binding cassette transporters, and cholesterol esterification pathways collectively determine whether sterol is synthesized, imported, exported, stored, or catabolized [5,6]. Extrahepatic synthesis remains physiologically important, but the liver is the dominant clinical control node for LDL concentrations because it clears apoB-containing particles and regulates VLDL supply to the circulation.

### 3.2. Statins: Hepatic Mechanism

Statins inhibit hepatic HMG-CoA reductase, the rate-limiting enzyme of the mevalonate pathway and the principal control point for de novo cholesterol synthesis in the hepatocyte. Inhibition reduces intracellular sterol content, which relieves SCAP-mediated retention of SREBP2, allowing the transcription factor to activate LDLR gene expression. The resulting upregulation of hepatic LDL receptors increases the receptor-mediated endocytosis of LDL and other apoB-containing particles, lowering circulating LDL-C [2,9]. This mechanism is well-established across decades of pharmacokinetic, genetic, and clinical trial evidence and explains the proportional LDL-lowering observed with different statin potencies and doses.

Statins also reduce isoprenoid intermediates and thereby influence endothelial nitric oxide bioavailability, oxidative stress, leukocyte–endothelial interaction, and inflammatory signaling, although these pleiotropic effects should be understood as complementary to, and not substitutes for, LDL lowering [10,11].

### 3.3. Statin Safety: Proportional Framing for Clinical Use

Careful interpretation of adverse effects is warranted. Statin-associated muscle symptoms are a common reason for discontinuation, but randomized, double-blind trial evidence indicates that the absolute excess of muscle pain or weakness attributable to statins is small and concentrated mainly during the first year of therapy [12]. Consensus statements therefore recommend the structured assessment of symptom timing, creatine kinase levels, interacting medications, dose intensity, dechallenge, and rechallenge rather than attributing every symptom to statin exposure [13,14].

Whether statins accelerate sarcopenia is less settled. Observational and rehabilitation studies have reported impaired strength recovery or reduced sarcopenia-related quality of life in selected populations, whereas longitudinal community data and recent meta-analytic evidence have not shown a consistent overall increase in the incidence of sarcopenia [15,16,17,18]. Mechanistic reviews describe plausible pathways involving mitochondrial function, calcium handling, proteostasis, and mevalonate-derived intermediates, while also acknowledging potential anti-inflammatory and microvascular effects that could benefit muscle in other contexts [67]. The conclusion is individualized: statins can be clinically problematic for some frail or predisposed patients, but a universal statin-induced catabolic effect on skeletal muscle may not yet be established.

Glycemic effects are also measurable but generally acceptable. Meta-analyses show a small increase in incident diabetes, with somewhat greater risk under intensive-dose therapy or in populations already enriched for baseline insulin resistance [19,20,21]. Mechanistic and clinical studies also support changes in insulin resistance, insulin secretion, HbA1c, or related markers in susceptible individuals [22,23]. These trade-offs justify glucose monitoring and dose individualization in those with prediabetes, severe insulin resistance, frailty, or prior statin intolerance. In stable steatotic liver disease, available reviews support the safety of statins and their potential hepatic or cardiovascular benefits, reinforcing that hepatic inflammation should not be used as a reason to avoid indicated LDL-lowering therapy [68,69].

## 4. Gut Microbiota, Bile Acids, and Intestinal Sterol Loss

The gut microbiota modifies cholesterol homeostasis through several partially overlapping mechanisms. Bacterial bile salt hydrolase activity can increase deconjugation and fecal loss of bile acids, prompting hepatic cholesterol use for bile acid resynthesis. Other bacteria either incorporate cholesterol into bacterial membranes or convert cholesterol to coprostanol, a sterol that is less efficiently absorbed by the host [24,25,26]. Microbial short-chain fatty acids and secondary bile acids can also influence intestinal and hepatic transcriptional pathways governing lipid uptake, synthesis, transport, and enterohepatic recycling [27,28,29].

These mechanisms explain how microbiome composition can influence serum lipids without major changes in macronutrient intake. However, the modern gut microbiome is not uniformly cholesterol-lowering. Diet, host genetics, bile acid pool composition, medication and toxicant exposure, intestinal barrier function, and baseline metabolic phenotype all shape whether microbial effects are protective, neutral, or adverse. Thus, microbiome-targeted strategies should be framed as possible adjuncts rather than substitutes for established LDL-lowering therapies. Further research into probiotic supplementation may establish it as an adjunctive LDL-lowering therapy.

Comparing the microbiome’s influence on endogenous versus exogenous cholesterol metabolism is instructive. Bile salt hydrolase-expressing bacteria and coprostanol-converting organisms act on the shared enterohepatic bile acid pool regardless of dietary cholesterol intake. In a high-fat, high-cholesterol feeding model, gut microbiome composition was shown to mediate steatohepatitis progression through bile acid remodeling, acting downstream of dietary cholesterol load [30]. When dietary cholesterol is low and endogenous, hepatic synthesis is the primary source of sterol; microbial effects on bile acid pool composition remain active, but their net directional effect on serum LDL depends on baseline LDLR expression and hepatic synthesis rate. Direct human comparative data comparing microbiome contribution under matched low-exogenous versus high-exogenous cholesterol conditions remain limited and constitute a knowledge gap noted in Table 2.

**Figure 1:**
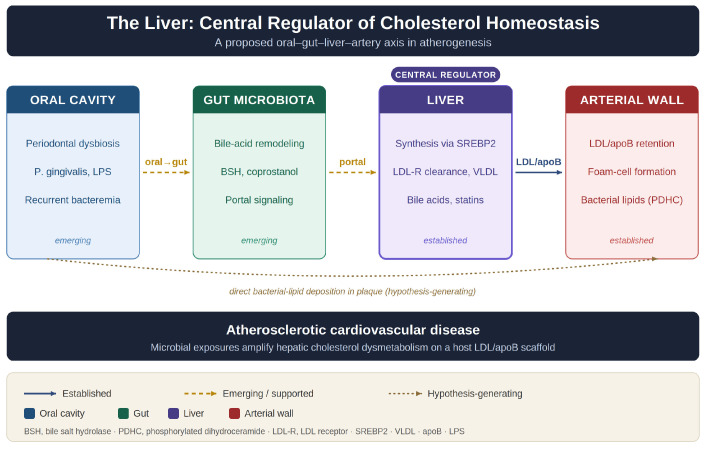
Proposed oral–gut–liver–artery axis linking oral dysbiosis, gut and portal signaling, hepatic inflammation, dyslipidemia, and plaque biology. Solid arrows indicate established or strongly supported pathways (H = human evidence; E = experimental evidence); dashed arrows indicate emerging or hypothesis-generating pathways.

This point is particularly relevant for the liver. All absorbed sterols and many microbial metabolites reach the liver through the portal circulation. High dietary cholesterol can worsen experimental steatohepatitis, in part through microbiota-mediated bile acid remodeling, illustrating that gut signals can modify hepatic cholesterol and inflammatory burden in a diet- and host-dependent manner [30].

## 5. Chronic Hepatic Inflammation and Intrahepatic Cholesterol Burden

Free cholesterol is not merely a passive passenger in fatty liver disease. Experimental and human-translational work implicates hepatic free cholesterol, oxysterols, lysosomal sterol trapping, Kupffer cell activation, and macrophage cholesterol overload in hepatocyte injury, stellate cell activation, and fibrotic progression [31,32,33]. The modern term MASLD captures the metabolic context of many cases previously categorized as nonalcoholic fatty liver disease (NAFLD); older studies cited here retain NAFLD or NASH terminology because those terms were used at publication.

Clinically silent liver inflammation could therefore alter cholesterol handling without producing a simple linear rise in serum total cholesterol. Inflammation may increase scavenger uptake, impair lysosomal sterol trafficking, alter SREBP-dependent synthesis, reduce effective bile acid disposal, promote the persistence of cholesterol crystals, and modify VLDL secretion. In some inflammatory states, serum cholesterol can fall while tissue lipid handling worsens; liver-specific sterol pathology may therefore be missed by routine lipid panels alone.

The hypothesis evaluated here is not that inflammation always causes hypercholesterolemia. It is that chronic low-grade hepatic inflammation, especially in metabolically susceptible hosts, may increase the intrahepatic burden of free or esterified cholesterol, destabilize hepatocyte and macrophage sterol processing, and favor a lipoprotein milieu that is more atherogenic than the serum cholesterol concentration alone would predict.

## 6. Oral–Gut–Liver Signaling and *Porphyromonas gingivalis*

Periodontitis is a chronic inflammatory reservoir with repeated access to the bloodstream and gastrointestinal tract. The oral–gut–liver axis has become increasingly relevant because swallowed oral pathobionts can alter gut ecology and barrier function, and because transient bacteremia and microbial products can reach the liver via systemic or portal routes [70,71]. *Porphyromonas gingivalis* is particularly relevant because it is a keystone periodontal pathogen with immune-evasive virulence factors, outer membrane vesicles, lipopolysaccharide, gingipains, and unusual bacterial sphingolipids.

### 6.1. Phosphorylated Dihydroceramides and Inflammatory Amplification

One of the most novel links between periodontal biology and vascular inflammation is the production of phosphorylated dihydroceramides (PDHCs), including phosphoglycerol and phosphoethanolamine dihydroceramide species, by *P. gingivalis* and related organisms. Foundational lipidomic work identified PDHC structures in *P. gingivalis* and demonstrated biological activity; subsequent studies recovered related lipids from diseased dental tissues and showed endothelial apoptosis and Toll-like receptor 2 signaling [34,35,36,37]. Critically, Nichols and colleagues demonstrated that phosphorylated dihydroceramides from common human bacteria are recoverable in human tissues, including atheroma-associated vascular specimens, providing direct physical evidence that bacterially derived lipids can enter and persist in host tissue compartments [38]. Bacterial sphingolipids need not be present in high abundance to exert biological effects, because their signaling potency at Toll-like receptor 2 and other innate immune receptors is substantial even at trace concentrations.

The conservative interpretation of the current evidence does not support the conclusion that bacterial sphingolipids constitute the bulk of the neutral lipid core of atherosclerotic plaque. The model is inflammatory amplification: low-abundance bacterial lipids, membrane fragments, extracellular vesicles, and biofilm components may alter endothelial activation, macrophage behavior, Toll-like receptor signaling, and plaque vulnerability on a host-derived LDL/apoB scaffold.

#### Bacterial Ceramides, Gut Bacteroidetes Sphingolipids, and Cardiovascular Disease

Ceramides have long been recognized as signaling mediators in atherosclerosis, and sphingomyelin is elevated in aortic plaque, but the question of bacterial origin adds an underappreciated dimension to their cardiovascular relevance. Bismuth and colleagues provided an early synthesis of the evidence that ceramide represents a common pathway in atherosclerotic disease, with roles in endothelial apoptosis, LDL retention, inflammatory amplification, and foam-cell formation [61]. More recently, spatial lipidomics of coronary atherosclerotic plaque has identified ceramides, phosphatidylcholines, and ether phospholipids as spatially organized components of plaque architecture in a familial hypercholesterolemia swine model, reinforcing the idea that lipid class composition, not just cholesterol concentration, matters for plaque vulnerability [62].

Nemati and colleagues showed that serine dipeptide lipids produced by Bacteroidetes bacteria are deposited and hydrolyzed in human arteries, and that their abundance correlates with the severity of atherosclerosis in carotid endarterectomy samples [63]. These serine dipeptide lipids, including Lipid 654 and Lipid 430, are structurally related to the sphingolipid class and are bioactive at concentrations encountered in diseased tissue. Olsen provided a focused synthesis of this line of evidence, concluding that commensal Bacteroidetes-derived lipids may represent an underappreciated atherogenic factor operating through innate immune and inflammatory pathways [64].

Johnson and colleagues demonstrated, using a rigorous experimental model, that sphingolipids produced by gut bacteria, specifically Bacteroidetes, enter host metabolic pathways and measurably affect ceramide and dihydroceramide pools in the host liver and intestine [65]. This finding has significant implications for the oral–gut–liver–artery axis: it means that the gut microbiome is not only a passive modifier of bile acid metabolism and cholesterol absorption, but also a direct source of bioactive sphingolipids that can enter hepatic lipid pools. Heaver, Johnson, and Ley synthesized the structural diversity and metabolic effects of gut-bacterial sphingolipids, documenting how Bacteroidetes-derived lipids interact with host ceramide biosynthesis and immune signaling pathways and influence intestinal homeostasis [66]. Taken together, these findings indicate that the microbiome’s lipid contribution to atherogenesis is not limited to secondary bile acid remodeling or short-chain fatty acid signaling; it also includes the direct delivery of ceramide-class lipids that can alter hepatic lipid handling and vascular inflammation.

The cardiovascular significance of ceramides is now well-established beyond the context of plaque biology. Elevated plasma ceramides predict cardiovascular events independently of LDL cholesterol, and specific ceramide species have been incorporated into clinical risk-refinement tools in certain settings. What the bacterial ceramide literature adds is a mechanistic bridge between microbial community composition and ceramide flux in host tissues. This is consistent with the broader oral–gut–liver–artery model advanced in this review, and it provides that model with a specific lipid-metabolic mechanism that is testable through combined microbial source tracing and sphingolipidomics in longitudinal human studies.

### 6.2. Liver Models and Metabolic Signaling

Human association studies and experimental models support a link between *P. gingivalis* and steatotic liver disease. Yoneda and colleagues first implicated periodontal *P. gingivalis* in NAFLD pathogenesis, and later work associated the organism with disease progression [39,40]. In an animal study, obese or high-fat-fed mice exposed to *P. gingivalis* showed worsened fatty liver progression, inflammation, and steatohepatitis-like histology, with reported involvement of pathways such as CD36-PPARgamma signaling [41].

Animal studies also support both direct and indirect oral–liver routes. Orally administered *P. gingivalis* has been detected in the mouse liver and shown to alter hepatic glycogen synthesis through IRS-1/Akt/GSK-3beta signaling [42]. Oral administration can also induce gut dysbiosis, impair barrier-related gene expression, increase endotoxemia, and increase bacterial DNA recovery in the liver [43,44,45]. More recent studies implicate ferroptosis, NF-kappaB activation, macrophage Nrf2-dependent clearance, and sphingolipid-associated insulin resistance in *P. gingivalis*- or periodontitis-associated liver injury [46,47,48,49].

The broader sphingolipid literature reinforces metabolic plausibility without proving oral microbial origin. In human cohorts, ceramide-to-dihydroceramide ratios and circulating dihydroceramide species have been associated with insulin resistance, diabetes susceptibility, cardiometabolic risk, and NAFLD severity [72,73,74,75,76]. Pharmacologic metabolic improvement has been associated with reduced circulating dihydroceramide levels in selected studies [77,78]. These findings indicate that dihydroceramide flux is clinically relevant in metabolic disease, while leaving unresolved the extent to which circulating or tissue PDHC signals are either microbial or host-derived.

### 6.3. Periodontal Disease, Inflammation, and the Atherogenic Triad

Across modern cardiovascular and microbiome research, there is strong, converging evidence that periodontal disease is not merely a localized oral condition but a systemic inflammatory network that influences atherosclerosis. Multiple mechanistic and review studies demonstrate that oral pathogens can directly enter the bloodstream through recurrent bacteremia, disseminate to vascular tissues, and activate immune-inflammatory cascades that promote arterial disease [8].

High-risk periodontal pathogens (including *Porphyromonas gingivalis*, *Aggregatibacter actinomycetemcomitans*, *Tannerella forsythia*, *Treponema denticola*, and *Fusobacterium nucleatum*) exacerbate the atherogenic triad through distinct but complementary mechanisms. First, these organisms promote ApoB-related atherogenic processes by altering hepatic lipid metabolism, increasing VLDL production, and elevating circulating ApoB-containing lipoproteins. Second, they induce endothelial dysfunction through cytokine-mediated inflammation and the upregulation of endothelial adhesion molecules. Third, they enhance intimal retention and lipoprotein binding, thereby accelerating plaque accumulation and the progression of atherosclerosis [8].

Chronic immune activation, characterized by elevated inflammatory mediators such as interleukin-6 (IL-6) and tumor necrosis factor-alpha (TNF-alpha), links oral infection to both hepatic dyslipoproteinemia and arterial wall inflammation. Consistent with this model, bacterial DNA and virulence factors from oral pathogens are detectable within atherosclerotic plaques, thereby reinforcing their contribution to the maintenance of vascular inflammation and disease progression [8].

## 7. Viridans Streptococci, *Streptococcus anginosus*, and Plaque Inflammation

Viridans streptococci are abundant oral colonizers and recognized causes of transient bacteremia after toothbrushing, chewing, scaling, and other routine oral activities, especially when gingival inflammation is present [50,51,52]. *Streptococcus anginosus* group organisms are oral and gastrointestinal commensals with biofilm-forming capacity, tissue invasiveness, association with abscesses, and defined virulence factors [53]. These traits make them plausible inflammatory cofactors at vascular and hepatic interfaces.

Earlier studies detected periodontal and other bacterial signatures in human atheromatous tissue, and subsequent microbiome studies have shown overlap among oral, gut, and plaque microbial communities [54,55,56,57]. A landmark contribution to the field of bacterial origin of early arterial inflammation came from the INVEST study (Oral Infections and Vascular Disease Epidemiology Study), in which Desvarieux and colleagues demonstrated that the periodontal microbiota, specifically the subgingival burden of pathogenic species, was independently associated with carotid intima-media thickness in a large community-based cohort [58]. Ott and colleagues used the broad-range 16S rRNA gene analysis of bacterial communities from coronary atherosclerotic lesions of patients with coronary heart disease and documented diverse bacterial DNA signatures representing Proteobacteria, Actinobacteria, Bacteroidetes, and Firmicutes [56]. The Bacteroidetes representation in those coronary specimens is particularly relevant given what we now know about Bacteroidetes-derived serine dipeptide lipids and their deposition in human arteries [63].

Recent human studies provide stronger support for microbial participation in plaque biology. In the SCAPIS cohort, gut *Streptococcus* abundance was linked to subclinical coronary atherosclerosis [59]. A 2025 study reported the presence of viridans streptococcal DNA and biofilm localization in coronary and carotid specimens, with signals associated with severe disease and rupture-related inflammatory pathways [60]. These observations remain vulnerable to confounding by oral health, medications, metabolic syndrome, and shared inflammatory environments, but they strengthen the case for recurrent oral or enteric microbial stimulation as a contributor to plaque inflammatory phenotype.

The experimental work is consistent with this model. *P. gingivalis* can promote macrophage foam-cell formation, and diverse bacteria can induce Toll-like receptor-dependent lipid body formation in macrophages [79,80,81]. In animal studies, *P. gingivalis* accelerates atherosclerosis in *ApoE*-deficient mice and has recently been linked to plaque instability through lipid-laden macrophage necroptosis [82,83]. These findings support the inflammatory acceleration of plaque progression and destabilization, rather than the bacterial replacement of host-derived plaque lipids [84].

## 8. What Is Established, Emerging, and Hypothesized

The proposed model is strongest when its evidentiary layers are kept separate. Confidence categories in Table 1 were assigned based on four criteria: (1) consistency of findings across independent study groups; (2) evidence from human versus animal versus in vitro systems; (3) availability of a plausible mechanistic explanation supported by primary data; and (4) directionality established by intervention or genetic evidence rather than association alone. These are the authors’ narrative judgments, not GRADE classifications.

## 9. Proposed Oral–Gut–Liver–Artery Model and Knowledge Gaps

Figure 1 presents the model, with solid arrows indicating established or strongly supported pathways, and dashed arrows indicating emerging or hypothesis-generating links. Arrows are labeled ‘H’ when the pathway is primarily supported by human clinical or epidemiological evidence, and ‘E’ when support is primarily from experimental (animal- or cell-based) studies. A legend is provided below the figure. The model is intentionally nonlinear: oral inflammation, gut ecology, hepatic sterol handling, host metabolic context, and plaque inflammation can feed back on one another.

In metabolically susceptible hosts, recurrent periodontal inflammation may increase exposure to microbial ligands, bacterial extracellular vesicles, PDHCs, and transient bacteremia. Oral pathobionts swallowed may also perturb intestinal communities, barrier function, and bile acid signaling. These routes converge on the liver via the portal and systemic circulations, where Kupffer cells, hepatocytes, stellate cells, and recruited macrophages respond via Toll-like receptor, NF-kappaB, oxidative stress, sphingolipid, ferroptotic, and lysosomal stress pathways.

This model has several translational gaps. First, the detection of microbial DNA, PDHCs, or bacterial antigens in plaque does not establish viability, directionality, or causal contribution. Second, reverse causation is plausible: inflamed or lipid-rich plaque and metabolically injured liver may be permissive niches for microbial products rather than consequences of them. Third, metabolic syndrome, diet, diabetes, smoking, socioeconomic factors, oral hygiene, and medication exposure can confound associations among oral, gut, liver, and vascular tissues. Fourth, murine lipid metabolism and experimental pathogen dosing may differ from chronic human exposure. These limitations do not invalidate the model; they define the next studies needed to test it.

Table 2 maps each knowledge gap corresponding to a dashed-arrow pathway in Figure 1 to a proposed study design, primary endpoint, and minimum design feature needed to establish directionality.

## 10. Clinical Implications

In clinical practice, the model supports broader residual-risk thinking without weakening the central role of LDL-lowering. Hypercholesterolemia, MASLD, insulin resistance, recurrent periodontal disease, and accelerated atherosclerosis should not be managed as isolated silos when they coexist. Oral health evaluation, periodontal treatment, liver phenotype assessment, glycemic risk monitoring, and lipid therapy adherence may all influence the inflammatory and metabolic terrain in which atherosclerosis develops.

However, the potential value of microbial or periodontal interventions is adjunctive: reducing inflammatory exposure, improving gut–liver signaling, and possibly modifying the inflammatory phenotype of plaque. Prospective trials should test whether periodontal therapy, oral microbiome modulation, or targeted gut interventions improve hepatic inflammatory markers, bile acid profiles, sphingolipid signatures, lipoprotein kinetics, or statin responsiveness in well-phenotyped patients.

## 11. Conclusions

Most clinically relevant cholesterol flux is endogenous, and the liver is the principal organ determining whether cholesterol is synthesized, retained, exported, converted to bile acids, or cleared from plasma. Statins lower LDL by acting at this hepatic control node; their cardiovascular benefit remains robust, even though muscle symptoms, heterogeneous effects on muscle function, and modest glycemic trade-offs justify individualized monitoring in susceptible patients.

Gut bacteria can reduce or redistribute cholesterol through bile salt hydrolase activity, coprostanol conversion, and bile acid remodeling, but dysbiosis can also worsen hepatic inflammation and metabolic risk. Chronic low-grade liver inflammation may increase intrahepatic cholesterol burden and atherogenic lipoprotein handling, especially in MASLD and insulin-resistant states. Oral pathogens, particularly *P. gingivalis*, can serve as plausible inflammatory triggers via bacteremia, oral–gut translocation, hepatic immune activation, and bioactive bacterial sphingolipids such as PDHCs. Bacteroidetes-derived serine dipeptide lipids have been physically detected in human arteries, and gut bacterial sphingolipids demonstrably enter host ceramide pools through metabolic pathways, extending the microbial lipid contribution beyond *P. gingivalis*-specific chemistry to the broader Bacteroidetes community resident in both the oral cavity and gut. Viridans streptococci, including *S. anginosus* group organisms, may further contribute as recurrent bacteremic and plaque-associated inflammatory cofactors.

The most current postulate is an oral–gut–liver–artery axis in which microbial exposures amplify hepatic cholesterol regulation and the role of host apoB lipoproteins in soft-plaque formation. Future studies should integrate periodontal phenotyping, liver imaging, bile acid profiling, lipoprotein kinetics, sphingolipid lipidomics, bacterial lipid source-tracing using stable isotope and spatial lipidomic methods, and longitudinal cardiovascular outcomes within the same cohorts. The detection of Bacteroidetes-derived serine dipeptide lipids in human arteries and the demonstrated entry of gut bacterial sphingolipids into host ceramide pools define a high-priority mechanistic gap: we do not yet know the quantitative contribution of bacterially derived ceramide-class lipids to plaque ceramide content, nor whether that contribution is clinically modifiable through periodontal or microbiome interventions. Human evidence directly linking exposure to oral pathogens to increased hepatic cholesterol synthesis or LDL receptor suppression is currently unavailable. This body of evidence nonetheless supports an integrated model of atherogenesis in which hepatic cholesterol handling, ApoB-containing lipoprotein dynamics, vascular inflammation, and chronic microbial lipid deposition are biologically interconnected processes.

## Figures and Tables

**Table 1:** Evidence-strength summary for the proposed oral–gut–liver–artery model.

Domain	Confidence	Supported Conclusion	Key Limitation	Knowledge Gap	Key References
Liver as the control node	Established	Sterol sensing, LDLR expression, VLDL export, bile acid synthesis, and biliary disposal place the liver at the center of circulating LDL regulation.	Extrahepatic synthesis remains biologically important.	Better noninvasive measures of liver-specific sterol flux.	[1,2,3,4,5,6]
Statin mechanism	Established	HMG-CoA reductase inhibition lowers hepatic cholesterol and increases LDL receptor-mediated apoB particle clearance.	Mechanistic uncertainty is low; tolerance and adherence are the main challenges.	Phenotype-specific tolerability and adherence strategies.	[2,9,10,11]
Statin muscle and glycemic trade-offs	Moderate-to-strong	True statin-attributable muscle symptoms are usually uncommon; diabetes risk is modest and enriched in susceptible patients.	Sarcopenia and functional outcomes vary across populations and study designs.	Frailty-stratified trials and long-term function measures.	[12,13,14,15,16,17,18,19,20,21,22,23]
Gut microbiota and cholesterol	Moderate	Bile salt hydrolase activity, coprostanol conversion, and bile acid remodeling can alter absorption, fecal sterol loss, and hepatic signaling.	Effects vary by diet, host genetics, medications, and microbiome composition.	Reproducible microbiome interventions with clinically meaningful effects on lipids.	[24,25,26,27,28,29,30]
Hepatic inflammation and intrahepatic cholesterol	Moderate-to-strong	Experimental steatohepatitis supports free cholesterol loading, lysosomal stress, Kupffer cell activation, and fibrosis coupling.	Human studies often rely on serum lipids rather than direct hepatic sterol measurements.	Integrated liver imaging, bile acid profiling, and lipoprotein kinetics.	[31,32,33]
*P. gingivalis* and PDHC signaling	Moderate	PDHCs are bioactive, recoverable from diseased tissues, and linked to endothelial and Toll-like receptor signaling.	The relative contributions of microbial versus host sphingolipids are not quantified.	Isotope/source-tracing and human longitudinal lipidomics.	[34,35,36,37,38,39,40,41,42,43,44,45,46,47,48,49]
Viridans streptococci and *S. anginosus*	Emerging	Bacteremia, biofilm capacity, and plaque-associated signals support an inflammatory cofactor role.	Specific effects on human hepatic cholesterol production remain unproven.	Paired oral, blood, liver, and plaque microbiome studies.	[50,51,52,53,54,55,56,57,58,59,60]
Bacterial lipids as plaque lipid	Weak	Current evidence does not support replacing host apoB cholesterol as the dominant plaque lipid substrate.	Plaque-associated microbial molecules may retain high bioactivity even at low abundance.	Quantitative plaque source-tracing and spatial lipidomics.	[38,61,62,63,64,65,66]

**Table 2:** Knowledge gaps and proposed study designs for validating dashed-arrow pathways in Figure 1.

Dashed-Arrow Pathway	Gap	Proposed Study	Primary Endpoint	Minimum Design Feature
Oral *P. gingivalis* to hepatic lipid dysmetabolism	No human evidence that exposure to oral pathogens suppresses hepatic LDLR or increases VLDL synthesis.	Periodontal intervention trial (*n* ≥ 100): periodontal treatment vs. delayed control, with pre- and post-treatment apoB kinetics and liver steatosis imaging.	Fractional catabolic rate of LDL apoB; hepatic steatosis by the controlled attenuation parameter.	Randomized; standardized lipid-lowering therapy; 6-month follow-up.
Bacteroidetes serine dipeptide lipids to arterial wall accumulation	Deposition documented; whether quantity is clinically modifiable is unknown.	Longitudinal cohort: paired gut WGS, fasting serum serine dipeptide lipid profiling, coronary CTA at baseline and 3 years.	Serine dipeptide lipid serum levels correlated with coronary plaque progression by CTA.	Minimum 200 participants; fasting samples; blinded image analysis.
Gut bacterial sphingolipids to host ceramide pools	Demonstrated in rodent models; human quantitative contribution unknown.	Stable isotope tracing: oral gavage with ^13^C-labeled Bacteroides sphingolipids in volunteers; track label incorporation into plasma ceramides.	Isotope enrichment in plasma ceramide fractions 2–24 h post-dose.	GC-MS or LC-MS/MS; ethics-approved dose escalation.
Endogenous vs. exogenous cholesterol and microbiome contribution	No direct human comparison under matched low vs. high dietary cholesterol intake.	Crossover feeding study with controlled exogenous cholesterol; gut WGS, bile acid profiling, and LDL kinetics in each condition.	Bile acid pool composition and LDL fractional catabolic rate under each dietary condition.	Minimum 4-week washout; paired design; isotope kinetics preferred.
Oral dysbiosis to plaque inflammatory phenotype	Plaque microbial signals documented; whether oral-origin organisms drive a rupture-prone phenotype is unproven.	Paired subgingival, gut, and endarterectomy specimen microbiome plus spatial lipidomics in patients undergoing carotid endarterectomy.	Correlation of oral pathobiont abundance with plaque ceramide content and inflammatory gene signature.	*n* ≥ 50 endarterectomy cases; fresh-frozen and FFPE matched specimens.

## Data Availability

No new data were created or analyzed in this narrative review. Data sharing is not applicable to this article.

## Abbreviations

The following abbreviations are used in this manuscript:

apoB	Apolipoprotein B
ABC	ATP-binding cassette
ASCVD	Atherosclerotic cardiovascular disease
HMGCR	3-Hydroxy-3-methylglutaryl-coenzyme A reductase
LDL	Low-density lipoprotein
MASLD	Metabolic dysfunction-associated steatotic liver disease
NAFLD	Nonalcoholic fatty liver disease
NASH	Nonalcoholic steatohepatitis
PDHC	Phosphorylated dihydroceramide
SAMS	Statin-associated muscle symptoms
SREBP2	Sterol regulatory element-binding protein 2
TLR	Toll-like receptor
VLDL	Very-low-density lipoprotein
